# Study on clinical outcomes between non-transecting urethroplasty and lingual mucosal urethroplasty for iatrogenic bulbar urethral stricture treatment

**DOI:** 10.1186/s12610-023-00185-z

**Published:** 2023-05-04

**Authors:** Wei Le, Denglong Wu, Chengdang Xu, Weidong Zhou, Chao Li

**Affiliations:** Department of Urology, Tongji Hospital, Tongji University School of Medicine, 200065 Shanghai, China

**Keywords:** Bulbar urethral stricture, Iatrogenic, Urethral reconstruction, Non-transecting urethroplasty, Sténose urétrale bulbaire, Iatrogène, Reconstruction urétrale, Urétroplastie sans transsection

## Abstract

**Background:**

This study aimed to compare the clinical outcomes of non-transecting urethroplasty and lingual mucosal urethroplasty in the treatment of iatrogenic bulbar urethral stricture.

**Results:**

A total of 25 patients with iatrogenic bulbar urethral stricture were enrolled, 12 of whom underwent lingual mucosal urethroplasty, 13 patients who underwent non-transecting urethroplasty. All patients were followed-up and evaluated at 3 postoperative months. Evaluations included urethrography, maximum urine flow rate (Qmax), nocturnal erectile function testing, International Index of Erectile Function (IIEF-5) assessment, and Anxiety Related Scale (SAS) assessment. In terms of operation time, there was a significant difference between non-transecting urethroplasty and lingual mucosal urethroplasty. However, there was no significant intergroup difference in intraoperative blood loss. Both techniques were associated with significantly improved Qmax relative to preoperative rates, but there was no significant difference between the groups in this regard over 3 months of postoperative follow-up. Nocturnal penile tumescence and rigidity results showed that there was no significant change in tip hardness after surgery in the non-transecting urethroplasty group. Moreover, IIEF-5 scores indicated that there was no significant intergroup difference in terms of subjective postoperative erectile function. According to the preliminary psychological evaluations during postoperative follow-up, the anxiety scores of patients undergoing non-transecting urethroplasty significantly improved, but there was no significant change in the mean SAS score among patients who underwent lingual mucosal urethroplasty.

**Conclusion:**

Both surgical methods can achieve the clinical goal of treating iatrogenic bulbar urethral stricture. Non-transecting urethroplasty has the characteristics of short operation time, relative technical simplicity, and retention of the original erectile function of most patients, and the surgical outcomes of non-transecting urethroplasty are not inferior to those of lingual mucosal urethroplasty, and it is a promising technique for widespread use to treat bulbar urethral strictures.

## Résumé

**Contexte:**

Cette étude visait à comparer les résultats cliniques de l’urétroplastie non transectante et de l’urétroplastie avec greffe de  muqueuse linguale dans le traitement de la sténose urétrale bulbaire iatrogène. Un total de 25 patients présentant une sténose urétrale bulbaire iatrogène a été recruté, dont 12 ont subi une urétroplastie avec greffe de muqueuse buccale et 13 une urétroplastie non-transectante. Tous les patients ont été suivis et évalués à 3 mois postopératoires. Les évaluations comprenaient une uréthrographie, le débit urinaire maximal (Qmax), un test nocturne de la fonction érectile, l'évaluation de l'index international de la fonction érectile (IIEF5) et une évaluation de l'échelle d'anxiété.

**Résultats:**

En termes de durée opératoire, il y avait une différence significative entre l'urétroplastie non-transectante et urétroplastie avec greffe de muqueuse buccale. Cependant, il n'y avait pas de différence significative entre les groupes en ce qui concerne la perte de sang peropératoire. Les deux techniques ont été associées à une amélioration significative du Qmax par rapport aux taux préopératoires, mais il n'y avait pas de différence significative entre les groupes à cet égard sur 3 mois de suivi postopératoire. Les résultats de la tumescence et de la rigidité nocturnes du pénis ont montré qu'il n'y avait pas de changement significatif de la dureté de l'extrémité du pénis après l'opération dans le groupe d'urétroplastie sans transsection. De plus, les scores IIEF-5 ont indiqué qu'il n'y avait pas de différence significative entre les groupes en termes de fonction érectile subjective postopératoire. Selon les évaluations psychologiques préliminaires au cours du suivi postopératoire, les scores d'anxiété des patients ayant subi une urétroplastie non-transectante se sont améliorés de manière significative, mais il n'y a pas eu de changement significatif du score moyen de l’échelle d’anxiété chez les patients ayant subi une urétroplastie avec greffe de muqueuse buccale.

**Conclusions:**

Les deux méthodes permettent d'atteindre l'objectif clinique du traitement de la sténose urétrale bulbaire iatrogène. L'urétroplastie sans transsection présente les caractéristiques suivantes: temps d'opération court, simplicité technique relative et maintien de la fonction érectile initiale chez la plupart des patients. Les résultats chirurgicaux de l'urétroplastie sans transsection ne sont pas inférieurs à ceux de l’urétroplastie avec greffe de muqueuse buccale et cette technique est prometteuse pour une utilisation généralisée dans le traitement des rétrécissements urétraux bulbaires.

## Background

The treatment of bulbar urethral strictures is a complicated clinical problem [1]. In addition to riding injury, iatrogenic factors (such as intravenous catheterization, minimally invasive transurethral surgery (such as TURP, TURBT, ureteroscopy and lithotripsy) are among the common causes of bulbar urethral strictures [2].

Most bulbar urethral strictures caused by riding injuries are serious, and sometimes they may even form armor scars around narrow urethras. Compared with urethral strictures caused by riding injury, iatrogenic urethral strictures are associated with shorter narrow segments and less scarring.

Endoscopic urethral incision for urethral strictures longer than 1 cm is often unsatisfactory, and open urethroplasty is often required. There are many open surgical methods available for the treatment of bulbar urethral strictures. Commonly used surgical methods include free mucous membrane replacement urethroplasty techniques, such as those that use mucous membrane from the oral cavity, as well as bulbar urethral end-to-end anastomosis.

In many domestic urethral reconstruction centers, the popular surgical approach to treating bulbar urethral strictures is to use free oral mucosa to replace the urethra. The main surgical techniques include buccal mucosal or lingual mucosal urethral reconstruction [3, 4]. Free lingual mucosa is widely used for major urethral reconstruction centers in China. When the lingual mucosa is extracted by this method, surgical trauma is inflicted on the tongue. This affects postoperative lingual sensory and motor activity and (consequently) speech. Many patients will feel apprehensive about these complications and will find it difficult to consent to undergoing such procedures.

Although traditional bulbar urethral end-to-end anastomosis has a high success rate, the corpus spongiosum needs to be completely transected before anastomosis, and this extensive surgical trauma may damage the blood supply and innervation to the patient’s bulbar corpus spongiosum [5], potentially causing a series of complications and adverse outcomes, including impaired sexual function [6, 7].

In view of this problem, some scholars have proposed and applied the surgical method of urethral spongiform non-transecting anastomosis to treat bulbar urethral strictures, which can achieve better clinical outcomes [8].

To our knowledge, no published studies have compared the clinical outcomes of non-transecting urethroplasty with those of lingual mucosal urethroplasty. We attempted to treat iatrogenic bulbar urethral strictures with non-transecting urethroplasty, and we compared bulbar urethral reconstruction with lingual mucosal urethroplasty. By comparing clinical variables and patient outcomes associated with the two surgical procedures, we evaluated whether non-transecting urethroplasty could be used as a routine treatment option or a beneficial supplement for treating iatrogenic bulbar urethral strictures. Overall, we evaluated the technique’s value in the clinical treatment of iatrogenic bulbar urethral strictures.

## Methods

All study data were collected from 25 patients with bulbar urethral stricture in Tongji Hospital, which is affiliated with Tongji University, from 2010 to 2021. The study was conducted in accordance with the Declaration of Helsinki (as revised in 2013). The study was approved by the Ethics Committee Board of Tongji Hospital Affiliated to Tongji University (NO. K-KYSB-2020-0) and individual consent for this retrospective analysis was waived.

### Patient data

In our patients who chose to undergo non-truncating urethroplasty, the strictures were all less than 2 cm in length, and the distal urethra was separated as it approached the root of the penis to increase urethral freedom and reduce anastomotic tension. The inclusion criteria were as follows: (1) bulbar urethral stricture caused by various iatrogenic factors; (2) the presence of bulbar urethral stricture indicated by preoperative urethrography and soft cystoscopy; (3) the length of the stenosis was < 2 cm; (4) patients aged 15–50 years; (5) preoperative androgen (T), estradiol (E2), and prolactin (PRL) levels within the normal range; and (6) no penile blood flow reduction diagnosed by preoperative penile Doppler ultrasonography.

The exclusion criteria are as follows: (1) congenital urethral stricture; (2) patients with urethral stricture aged < 15 years or > 60 years; (3) complex urethral stricture—for example, the length of the stricture was ≥ 2 cm or more than two urethral strictures were observed; (4) patients with a history of two or more procedures involving urethral incisions; (5) patients with severe erectile dysfunction (penile head hardness < 20% detected by preoperative nocturnal penile tumescence and rigidity (NPTR) evaluation; and (6) patients with abnormal sex hormone levels.

### Preoperative preparation

The presence of bulbar urethral stricture was confirmed by urethrography and soft cystoscopy, and the length of the urethral stricture was estimated by urethrography. Antibiotic therapy, maximum urine flow rate (Qmax) testing, and International Index of Erectile Function (IIEF-5) assessments were administered before surgery, and patients with normal urinary function and negative urine culture results were considered suitable for surgery.

### Operative techniques

#### Lingual mucosal urethroplasty

**Fig. 1:**
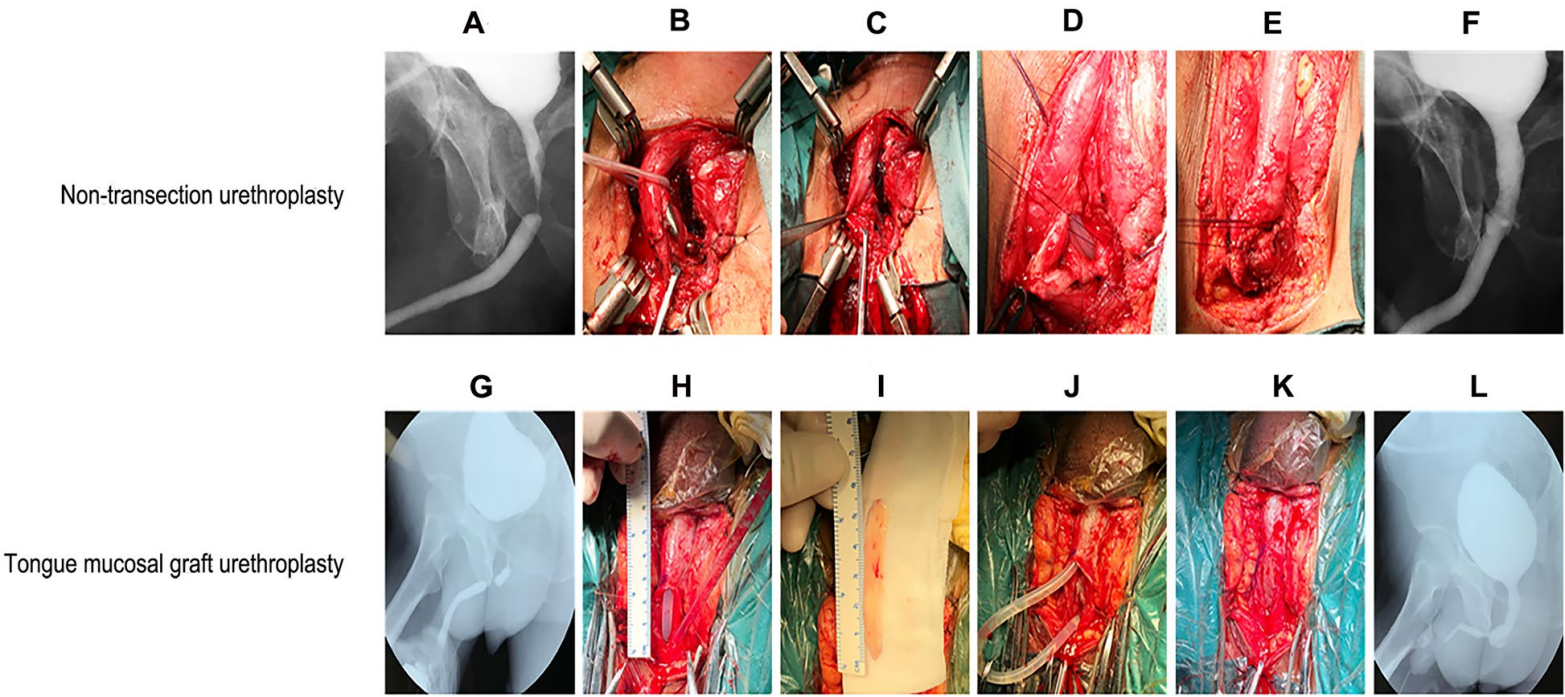
Preoperative and postoperative urethrography and surgical procedures of the two surgical methods. Bulbar corpus spongiosum non- transecting urethroplasty: **A** Preoperative urethrography: bulbar urethral stricture; **B** Dissociate and split the narrow urethra to expose the distal and distal normal urethra mucosa; **C** 3 − 0 absorbable suture of proximal urethral mucosa and indwelling catheter; **D** 3 − 0 suture the proximal end of absorbable line with the distal urethral mucosa; **E** Tighten and tie the distal and distal urethral mucosal sutures; **F** Postoperative urethrography: the lumen of the bulbar urethra was unobstructed without stenosis lingual mucosal bulbous urethroplasty: **G** Preoperative urethrography: bulbar urethral stricture; **H** Dissociate and split the narrow urethra to expose the distal and distal normal urethral mucosa; **I** The mucous membrane of the tongue was taken and trimmed; **J** Urethra was dissected and the lingual mucosa Inlay was used to reconstruct the dorsal urethra; **K** Urethra was dissected and the lingual mucosa Onlay was used to reconstruct the ventral urethra; **L** Postoperative urethrography: the lumen of the bulbar urethra was unobstructed without stenosis

(1) An inverted “Y” incision was made at the perineum (2). Then the bulbar corpus spongiosum was separated (3). A urethral dilator was inserted into the anterior urethra to locate the position of the urethral stricture and longitudinally open the stenosed segment to the extent of the normal distal and proximal urethral urethral mucosa (4). With the goal of maintaining the corpus spongiosum’s continuity, the rigid scar tissue constituting the urethral stricture was removed, and the urethral incision length was measured (5). Then the patient’s oral cavity was cleaned and disinfected. After the tongue tip was pulled and marked with a marker, an incision, about 2 cm in width, was made. The length of the incision was determined according to the length of urethral incision, and the excised mucosa was used for ventral onlay reconstruction of the bulbar urethra. If the stenosis was severe, the length of tongue mucosa removed was equivalent to about twice the length of the resected urethra, and the dorsal inlay combined with the ventral onlay was used to reconstruct the bulbar urethra. The excised lingual mucosa was placed in ice-cooled normal saline to prune the tissue (6). The free lingual mucosal tissue was dissected into the bulbar urethra, and the bulbar urethra was reconstructed using the ventral onlay method. If the stenosis was severe, the bulbar urethra was reconstructed using the dorsal inlay method combined with the ventral onlay method. Finally, (7) an indwelling 18 F silicone catheter was inserted (Fig. 1), and (8) the incision was closed layer by layer.

#### Non-transecting urethroplasty

Steps (1), (2), (3) and (4) are the same as those for lingual mucosal urethroplasty [5]. After the proximal and distal urethra was sutured intermittently at points 1, 2, 4, and 5 in the lithotomy position, an indwelling 18 F silicone catheter was inserted, and then the proximal and distal urethra was sutured at points 7, 8, 10, and 11 [6]. All sutures were tightened to bring the proximal and distal urethra close together, and each suture was tied separately to complete urethral anastomosis in the context of a corpus spongiosum that has not been resected [7]. The outer corpus spongiosum of the urethral anastomosis was reinforced and sutured again with 3 − 0 absorbable suture (Fig. 1) [8]. The incision was closed layer by layer.

#### Operative time and intraoperative blood loss

Surgical data of the two groups were collected, including surgical time and estimated blood loss. Surgical time was the time from the beginning of the operation (skin incision) to the end of surgery (skin closure). The amount of intraoperative blood loss was estimated by measuring the difference between the amount of blood collected by an intraoperative negative-pressure suction device and the weight of preoperative and postoperative hemostatic gauze.

#### Postoperative treatment and follow-up

All patients received prophylactic antibiotics for about 1 week. Catheters were removed after 3 postoperative weeks, and urethrography was performed 1 month later. Follow-up criteria were mainly assessed by preoperative and postoperative differences in NPT, IIEF-5 scores and psychological status. The criterion for recurrence of postoperative urethral stricture was a urinary flow rate < 15 ml/s.

#### Evaluation of postoperative sexual function–related indicators

Three months postoperatively, erectile function–related assessments (NPTR and IIEF-5) were conducted, and nocturnal erectile hardness was further analyzed using penile TIP data. The Rigiscan testing instrument usually measures the erectile hardness of the head of the penis (TIP) and the root of the penis (BASE) while the patient is sleeping to reflect the patient’s true level of erectile function. According to the current internationally recognized reference ranges, patients with tip hardness below 60% were considered to have organic erectile dysfunction. At the same time of NPTR measurement, IIEF-5 scale analysis was performed, and the scores obtained were statistically analyzed.

### Statistical analysis

PASW Statistics for Windows, version 18.0 (SPSS Inc. Chicago, IL, USA) was used to run independent-samples t-tests and paired t-tests for preoperative and postoperative Qmax, IIEF-5 scores, and NPRT data. A *P* value < 0.05 was considered statistically significant.

## Results

### Operation and postoperative urinary outcomes

**Fig. 2:**
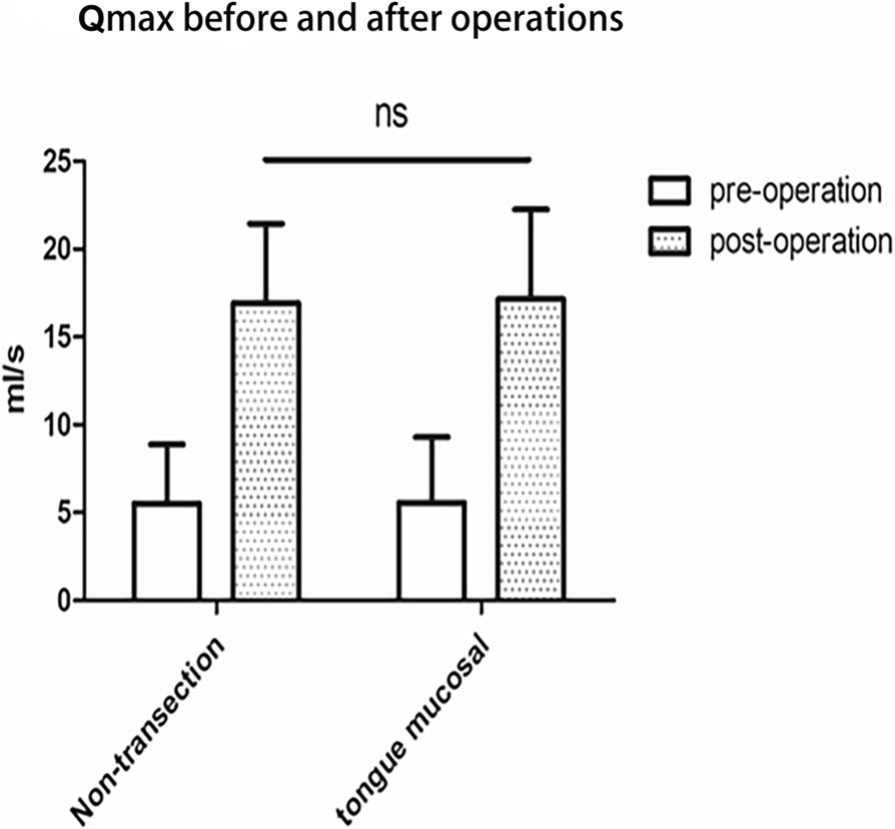
Changes in parameters of maximum postoperative urine flow rate between the two surgical methods. Qmax of the two surgical methods was significantly improved after catheter removal, but there was no significant statistical difference in Qmax parameters between the two groups after catheter removal (NS: compared with the lingual mucosal urethral surgery group, *P* > 0.05)

There was one patient in each group with progressive dysuria after removal of the catheter. The patient who failed lingual mucosal urethroplasty has been regularly treated with urethral dilation to date, and the patient from the non-transecting urethroplasty group underwent a second open surgery (resection of the lower pubic margin, resection of urethral stricture, and end-to-end urethral anastomosis) 6 months after the initial operation; the catheter was removed 3 weeks after the second operation, and the urine flow was good. For all other patients, throughout follow-up, urethrography indicated that the original urethral stricture was cured and that the urethra was smooth (Fig. 1). The mean postoperative Qmax indices associated with the two techniques significantly improved between preoperative and postoperative assessments (lingual mucosal urethroplasty, 17.16 ± 5.11 ml/s vs. 5.58 ± 3.73 ml/s; non-transecting urethroplasty, 16.92 ± 4.53 ml/s vs. 5.54 ± 3.36 ml/s). In terms of postoperative Qmax, there was no significant difference between the two groups (*p* > 0.05) (Fig. 2).

### Surgical indicators

**Fig. 3:**
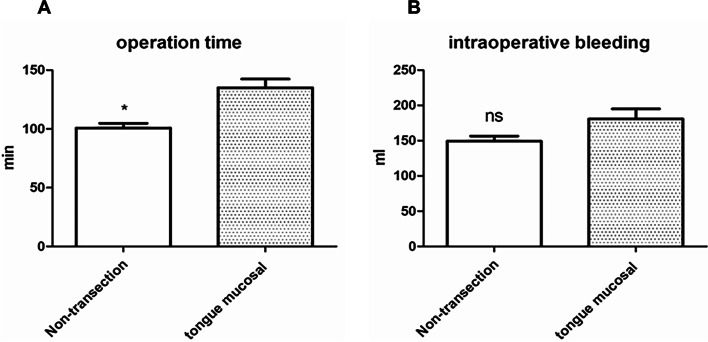
Comparison of operative time and intraoperative blood loss between the two surgical methods. **A** Operative time of the two surgical methods. The operative time of lingual mucosa urethroplasty was 135 ± 24.67 min; The operation time of non- transecting urethroplasty was 100.69 ± 14.48 min, and the operation time of non- transecting urethroplasty was shorter, with statistical difference (*: compared with the lingual mucosal urethral surgery group, *P* < 0.05). **B** Comparison of intraoperative blood loss between the two surgical methods. Intraoperative blood loss during lingual mucosal urethroplasty was 180 ± 47.16ml, the intraoperative blood loss during non- transecting urethroplasty was 149.2 ± 25.55ml, and there was no statistical difference between the two groups (NS: compared with the lingual mucosal urethral surgery group, *P* > 0.05)

The mean operative time associated with lingual mucosal urethroplasty was 135 ± 24.67 min; the mean operation time associated with non-transecting urethroplasty was 100.69 ± 14.48 min, and this difference was statistically significant (Fig. 3 A).

The mean intraoperative blood loss was 180 ± 47.16 ml in the lingual mucosal urethroplasty group, compared with 149.2 ± 25.55 ml associated with non-transecting urethroplasty; this difference was not statistically significant (Fig. 3B).

### Postoperative sexual function evaluation

**Fig. 4:**
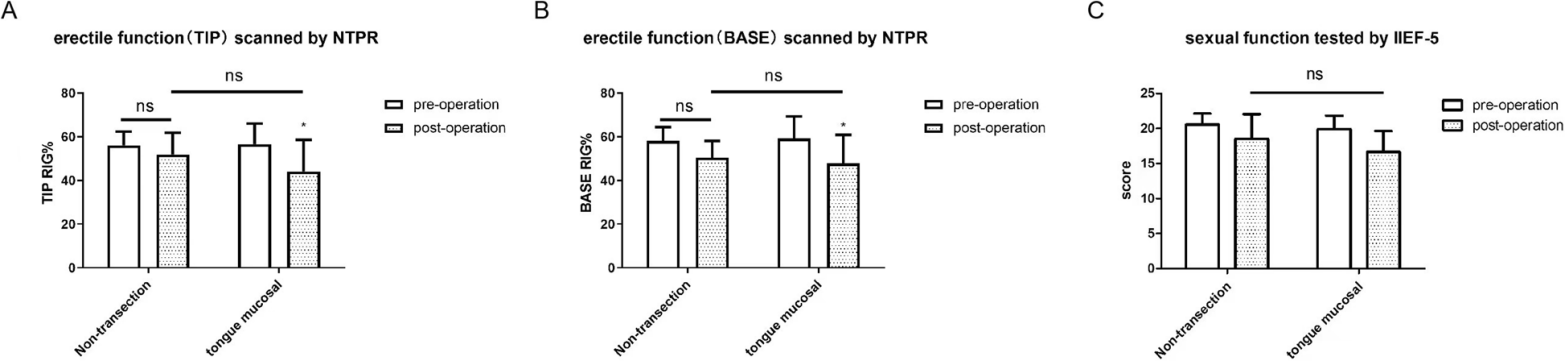
Penile tip hardness values of Post-operative NPTR analysis and IIEF-5 score results between the two surgical methods. **A** Penile head hardness values of Post-operative NPTR analysis. It showed a downward trend in TIP hardness values of the two different surgical methods, but there was no statistical difference in TIP hardness values of the non- transecting urethra group. There was statistically significant difference in the hardness decrease of the lingual mucosa group, but there was no statistically significant difference in the penile head hardness between the two groups after surgery. **B** IIEF-5 score results. Follow-up results 3 months after surgery indicated that iIEF-5 value decreased in both the lingual mucosa group and the non- transecting group, and the decrease was statistically significant (*P* < 0.05). There was no significant difference in IIEF-5 score between the two groups during postoperative follow-up (*P* > 0.05)

At the 3-months follow-up point after surgery, the mean NPTR rating for the lingual mucosal urethroplasty group was 42.91 ± 15.33% compared with 56.67 ± 9.42% before surgery (*p* < 0.05). The corresponding outcomes for the non-transecting urethroplasty group were 51.92 ± 9.91% after surgery vs. 56.15 ± 6.25% before surgery (*p* > 0.05). There was no significant difference in tip hardness parameters between the groups (*p* > 0.05) (Fig. 4 A, B).

In the lingual mucosal urethroplasty group the mean was IIEF-5 score was 19.91 ± 1.89 before surgery vs. 16.67 ± 2.98 after surgery (*p* < 0.05). The mean scores for the non-transecting urethroplasty group were 20.53 ± 1.59 and 18.53 ± 3.47, respectively (*p* < 0.05). There was no significant difference in mean IIEF-5 scores betwen the two groups during postoperative follow-up (*p* > 0.05) (Fig. 4 C).

### Preliminary evaluation results of postoperative psychological state

**Fig. 5:**
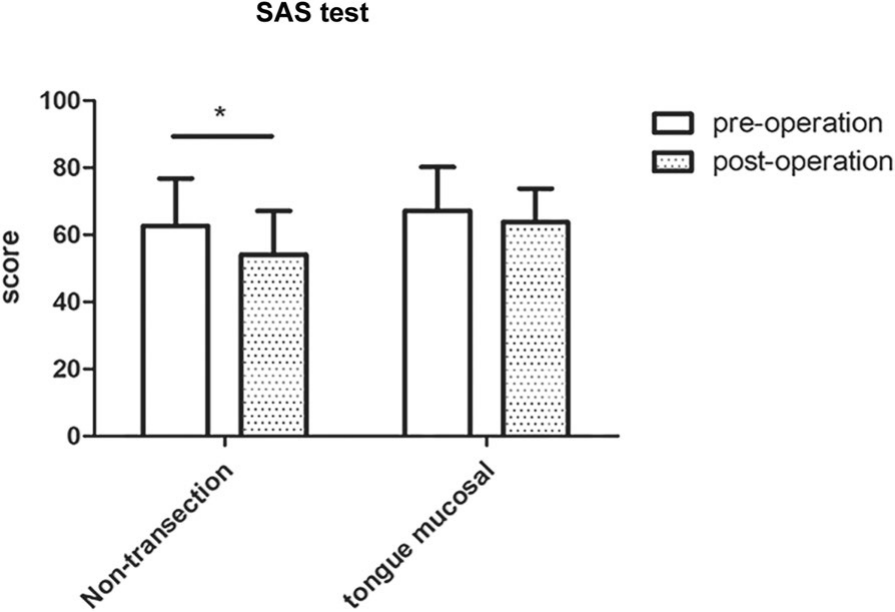
Postoperative anxiety psychological evaluation results of the two surgical methods. According to the follow-up of SAS anxiety score, the results showed that the postoperative anxiety SAS score of the non- transecting surgery group significantly decreased; There was no significant difference in the anxiety score of the lingual mucosa surgery group (*P* > 0.05), although the mean value was decreased. There was no significant difference in SAS score between two groups after operation (*P* > 0.05)

The mean statistics of anxiety score scale (SAS) for patients in the non-transecting urethroplasty group significantly improved 3 months after surgery (62.59 ± 14.18 before surgery vs. 54.13 ± 12.97 after surgery, *p* < 0.05). There was no significant change in the mean SAS score after surgery in the lingual mucosal urethroplasty group (preoperative 67.18 ± 13.06 vs. postoperative 63.75 ± 10.05, *p* > 0.05). There was no significant difference in node SAS scores between the two groups 3 months after surgery (*p* > 0.05) (Fig. 5).

## Discussion

Urethral stricture is still a common problem, which can arise from various causes. Iatrogenic causes, trauma, and idiopathic strictures are the main causes in the world today [9]. The bulbar urethra is the most common site of anterior urethral stricture formation. Currently, if the length of a stricture exceeds 1.0 cm, intraurethral incision should not be used for treatment, and open surgery should be used for bulbar urethral reconstruction, and this may be associated with clinical benefits in improving postoperative urination status [10, 11].

In addition to riding injuries, which have been considered the most common causes of bulbar urethral strictures, the incidence of iatrogenic bulbar urethral strictures is increasing with the increasing popularity of various minimally invasive transurethral operations and the widespread use of urethral catheters. Most bulbar urethral injuries caused by riding injuries are serious, and these sometimes may even form armor scars around the narrow urethra. Compared with urethral strictures caused by riding injury, the stenosis distance of patients with iatrogenic urethral strictures is shorter, and the associated scarring may also be less severe [12].

Traditional end-to-end bulbar urethral anastomosis is performed with one-stage anastomosis after the removal of spongy fibrosis and the narrowed urethra. For urethral strictures caused by riding injury, the urethral stricture segment is long and the fibrosis is severe. Therefore, traditional bulbar urethral end-to-end anastomosis with corpus spongiosum transection is an effective method for the treatment of this kind of stricture. However, a major disadvantage of the traditional end-to-end anastomosis is that the urethra must be completely transected, which may impair the penis blood supply and innervation [5]. Previous reports have shown that corpus spongiosum dissection has no significant negative impact on erectile function [6]. However, surgical cutting of nerve fibers traveling along the urethra can reduce the sensitivity of the glans and distal penis and lead to ejaculation dysfunction that may affect sexual activity [6]. Preservation of the distal urethral blood supply has also been shown to be crucial in the treatment of multiple penile urethral strictures [7].

Based on the above evidence, some scholars proposed a surgical method that does not require the complete transection of the corpus spongiosum [13, 14]. This operation can maximize the preservation of blood supply to the distal corpus spongiosum. Theoretically, it is possible to reduce the risk of postoperative erectile dysfunction or glans ischemia by minimizing penile blood supply interruption (due to urethral disconnection) and preserving the bulbar artery, which is conducive to follow-up urethral intervention; this technique has been performed in some clinical centers.

Most patients with iatrogenic bulbar urethral strictures have relatively short stenotic segments, and associated scarring may also be less severe; in such cases, only the stenotic part of the urethra and surrounding spongy fibrosis need to be removed for treatment. Therefore, non-transecting urethroplasty for the treatment of iatrogenic bulbar urethral strictures has good theoretical feasibility. Our center has also carried out non-transecting urethral surgery for treating urethral strictures at the bulbar membrane, and the results suggest that, compared with traditional end-to-end anastomosis, this method has certain advantages during the perioperative period and in terms of postoperative rehabilitation indicators [15].

In China, free graft replacement urethroplasty is also an option for the treatment of bulbar urethral strictures. The lingual mucosal epithelium is thick and rich in elastic fibers, and the lamella propria is thin and tough. The tissue has good elasticity and antimicrobial properties, which makes it suitable for survival in a wet environment. Therefore, this procedure is also used in the clinical treatment of long urethral strictures [16, 17]. The lingual mucosa is relatively convenient for sampling, and adult tongue mucosa samples can be as large as 6 cm × 2 cm on one side. Bilateral sampling can yield more tissue for urethral reconstruction and has also been associated with favorable clinical outcomes [18].

However, it must be pointed out that due to oral sampling, especially sampling of the lingual mucosa, this method can greatly impact psychological well-being and postoperative lingual sensation, motor function, and speech expression, and inevitably lead to certain negative results for patients’ postoperative rehabilitation. Based on these findings, we explored whether non-transecting urethral surgery could be a routine treatment option or beneficial supplement for iatrogenic bulbar urethral strictures.

At present, few published studies have compared the clinical outcomes of non-transecting urethroplasty with lingual mucosal urethroplasty. Therefore, in this study, patients with bulbar urethral strictures were enrolled to receive either of two surgical procedures, and their prognoses and a series of clinical variables were analyzed.

Urethroplasty takes a long time it requires the removal free tissue, but there was no difference in intraoperative blood loss between the two methods. Our results suggested that the time required for non-transecting surgery is shorter than that required for lingual mucoplasty. In terms of the recovery of postoperative urinary function, although one patient in each group had difficulty urinating after removal of the catheter, the rest of the patients underwent successful surgery, associated with satisfactory postoperative urinary function, according to postoperative urethrography. There was no significant difference in mean postoperative Qmax between the two surgical methods, suggesting that the non-transecting surgical method is also associated with favorable urinary outcomes.

We also conducted a follow-up analysis of sexual well-being. Via NPTR testing, we found that tip hardness in patients in the non-transecting urethroplasty group showed a decreasing (non-significant) trend postoperatively. In contrast, in the lingual mucosal urethroplasty group, tip hardness decreased significantly after surgery. These results suggest that the non-transecting operation may have a certain protective effect on the postoperative sexual function of patients with bulbar urethral strictures. However, the results need to be further clarified with larger-scale studies and clinical trials (e.g., ICI papaverine sponge injection with penile Doppler ultrasound).

The IIEF-5 scale was also used to observe that there was a downward trend in postoperative indices in both groups, with differences between preoperative and postoperative mean scores. A possible explanation is that the pain of the surgical incision, the discomfort of the oral cavity, and the psychological effects on patients have a certain negative impact on erectile function, which will affect sexual health. At the same time, our SAS evaluation may also confirm that postoperative anxiety normally exists in patients undergoing lingual mucosal urethroplasty, which may be associated with abnormal postoperative mastication and speech function. In contrast, postoperative anxiety improved significantly in patients who underwent non-transecting urethroplasty. However, conclusions regarding the long-term psychological rehabilitation and mood state of postoperative patients require long-term follow-up evaluation and multidimensional psychological evaluations.

In conclusion, urethral anastomotic repair with vascular preservation has certain functional advantages compared with traditional disconnection anastomotic repair for treating iatrogenic bulbar urethral strictures. Lingual mucosal urethroplasty is an invasive procedure, with surgical trauma having implications on the rehabilitation of patients’ psychological health and speech function. We conducted a systematic study on the treatment of iatrogenic bulbar urethral strictures via non-transecting urethroplasty and lingual mucosal urethroplasty. To our knowledge, no reports exist of such a comparison, so this study is novel. Our study findings suggest that non-transecting urethral surgery takes less time and can be associated with favorable urinary outcomes. Moreover, postoperative NPTR findings suggests that a considerable number of patients can retain their original sexual function, suggesting that this surgical method has a protective effect on sexual function. Additionally, the non-transecting urethroplasty group may have certain advantages in terms of postoperative psychological rehabilitation. It can be used as an alternative surgical method for treating clinical bulbar urethral strictures, and its postoperative outcomes are not inferior to those of lingual mucosal urethroplasty. Further clinical follow-up may be required to confirm that urination and sexual function outcomes are maintained over the long term.

## Data Availability

The datasets used and analysed during the current study are available from the corresponding author on reasonable request.

## Abbreviations

T: Preoperative androgen
E2: Estradiol
PRL: Prolactin
NPTR: Nocturnal penile tumescence and rigidity
Qmax: Maximum urine flow rate
IIEF-5: International Index of Erectile Function
SAS: Anxiety score scale
